# Tunable Temperature Response of a Thermochromic Photonic Gel Sensor Containing *N*-Isopropylacrylamide and 4-Acryloyilmorpholine

**DOI:** 10.3390/s17061398

**Published:** 2017-06-15

**Authors:** Hwanam Kye, Young Gook Koh, Youkyung Kim, Sung Gu Han, Hyunjung Lee, Wonmok Lee

**Affiliations:** 1 Department of Chemistry, Sejong University, 209 Neungdong-ro, Gwngjin-gu, Seoul 05006, Korea; ghkskadl@naver.com (H.K.); alsekddl@hanmail.net (Y.K.); chemiboy85@gmail.com (S.G.H.); 2 Engain Co. Ltd. Korea Bio Park BLD C-201, Seongnam 13488, korea; young.koh@engain.co.kr; 3 School of Advanced Materials Engineering, Kookmin University, 77 Jeongneung-ro, Seongbuk-gu, Seoul 02707, Korea

**Keywords:** temperature sensor, *N*-isopropylacrylamide, 4-Acryloyilmorpholine, lower critical solution temperature, opal templating, photonic gel

## Abstract

In this study, thermochromic photonic gels were fabricated using 2-hydroxyethyl methacrylate (HEMA) as a hydrogel building block, and 4-Acryloyl morpholine (ACMO) and *N*-isopropylacrylamide (NIPAAM) as thermoresponsive monomers with different critical solution temperature behaviors. Rapid photopolymerization of opal-templated monomer mixtures of varying ACMO contents formed five individual thermochromic inverse opal photonic gels integrated on a single substrate. With temperature variation from 10 °C to 80 °C, the changes in reflective colors and reflectance spectra of the respective thermochromic gels were noted, and λ_peak_ changes were plotted. Because NIPAAM exhibits a lower critical solution temperature (LCST) at 33 °C, the NIPAAM-only gel showed a steep slope for dλ_peak_/dT below 40 °C, whereas the slope became flatter at high temperatures. As the ACMO content increased in the thermochromic gel, the curve of dλ_peak_/dT turned out to be gradual within the investigated temperature range, exhibiting the entire visible range of colors. The incorporation of ACMO in NIPAAM-based thermochromic gels therefore enabled a better control of color changes at a relatively high-temperature regime compared to a NIPAAM-only gel. In addition, ACMO-containing thermochromic gels exhibited a smaller hysteresis of λ_peak_ for the heating and cooling cycle.

## 1. Introduction

Despite it being uncommon, there are some two-component liquid systems that exhibit a lower critical solution temperature (LCST) below which the mixed components are miscible. LCSTs are more often found in polymer solutions where a temperature rise has resulted in an unfavorable entropy of mixing [1]. Poly (*N*-isopropylacrylamide) (PNIPAAM) in water is one of the most studied examples of a polymer solution exhibiting LCST behavior; it is a swollen hydrogel below its LCST at 33 °C, while a temperature increase above this results in a reversible collapse transition due to the exposed isopropyl moieties. Poly (4-Acryloyl morpholine) (PACMO) has a higher LCST in water—this is due to morpholine being less hydrophobic [2]. ACMO is a vinyl monomer with hydrophilic and non-ionic characteristics; these properties make it ideally suited for increasing the swelling ratio of the hydrogels and facilitating water penetration into the polymeric network [3]. PACMO typically has an LCST at 88 °C, which is much higher than that of PNIPAAM. The derivatives from PACMO have a wide variety of use in peptide synthesis, enzyme immobilization, membranes for blood plasma separation, and drug release applications [4]. When it comes to “smart” drug delivery applications, the LCST of a material needs to be adjusted to be at near-body temperature [5,6,7]. The unique behavior of LCSTs in hydrogels also makes it possible for a temperature-based sensor to be fabricated around the reversible swelling-deswelling that occurs upon a change in temperature. Recently, there have been reports of thermochromic hydrogels utilizing temperature-driven volume changes in photonic crystal structures [8,9,10,11,12,13,14,15]; some studies have made use of this by applying an opal-templated PNIPAAM hydrogel in the fabrication of thermochromic sensors in order to induce reversible volume changes near the LCST of NIPAAM [8,9]. In addition, PNIPAAM hydrogels have also been recently used as force sensors [16]. 

Copolymerization of NIPAAM with other functional monomers has enabled a variety of dual sensors for factors such as temperature-pH [13,14], temperature-glucose [17], and temperature-light [10]. It should be noted, however, that the use of NIPAAM limits the possible temperature ranges to be around the LCST of NIPAAM; this means that there is a negligible shrinkage in volume at temperatures above 40 °C. The LCST phenomenon of a specific polymer system can be thus useful to develop a novel polymer thermometer [18,19]. In this study, noting that ACMO has a higher LCST than NIPAAM, those two thermoresponsive vinyl monomers are simultaneously incorporated within an opal template in order to investigate temperature-sensing photonic gel operating at high temperatures (i.e., above 50 °C).

## 2. Materials and Methods 

The following materials were purchased for the photo-polymerization of the photonic gel sensor and used without further purification: 2-hydroxyethyl methacrylate (HEMA (mol. mass. 130.14 g/mol), Junsei Chemical Co., Tokyo, Japan); *N*-isopropylacrylamide (NIPAAM (mol. mass. 113.16 g/mol), Sigma-Aldrich, St. Louis, MO, USA); 4-Acryloyl morpholine (ACMO (mol. mass. 141.17 g/mol), Sigma-Aldrich); *N,N’*-methylenebis(acrylamide) (MBAA, Sigma-Aldrich); and Irgacure-651 (Ciba Specialty Chemicals, Basel, Switzerland). Deionized (DI) water was supplied by a water purification system (Human Technologies, Utica, NY, USA) that had been equipped with ion exchange columns. 

**Figure 1:**
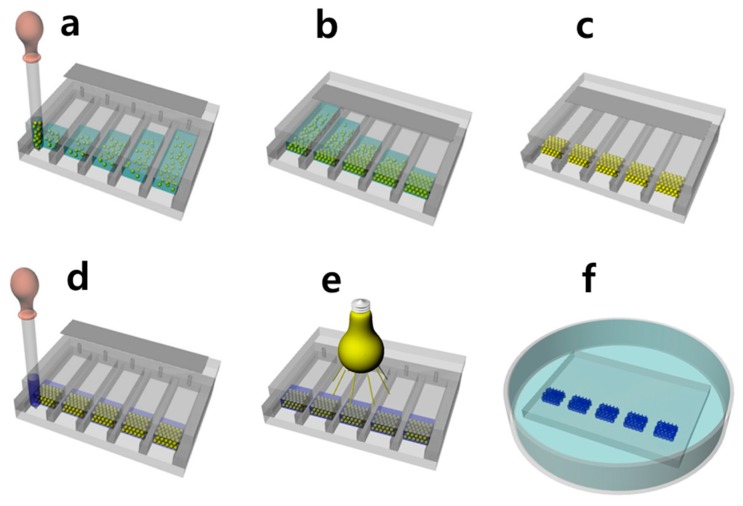
Schematic description for the fabrication procedure of the thermochromic photonic gel: (**a**) Infiltrating the aqueous dispersion of the PS in the five channels of the Directed Enhanced water Evaporation for Colloidal Assembly (DEECA) cell from the front slits with open pin-holes at the other ends; (**b**) Taping the pin-holes, and subsequently drying the water in order to induce colloidal crystallization at the fronts; (**c**) Annealing the dried PS opal templates; (**d**) Filling the precursor mixtures with different *N*-isopropylacrylamide (NIPAAM)/ 4-Acryloyl morpholine (ACMO) contents within the interstitial volume of the five opal templates; (**e**) UV lighte exposure and photopolymerization of the mixed monomers; (**f**) Etching away the PS templates in order to complete the fabrication of the five thermochromic photonic gels with controlled NIPAAM/ACMO contents.

The fabrication procedure for the templated polymerization of the hydrogel is described by the authors’ previous report [20] and is described schematically in Figure 1. The method is known as Directed Enhanced water Evaporation for Colloidal Assembly (DEECA), and it enables the rapid fabrication of opal template arrays within a few hours. Polystyrene (PS) microspheres with an average diameter of 230 nm and 235 nm (named PS-230 and PS-235, respectively) were polymerized by emulsion polymerization so as to be used as template particles [21]. Five 30-µm-thick opal template film arrays were fabricated by filling a custom-made DEECA cell, which contains five 60 mm × 5 mm channels, with ~10 wt % aqueous dispersions of PS-230 or PS-235 microspheres (Figure 1a) before crystallizing for 1 h (Figure 1b) and subsequently drying and annealing the opal films in an oven at 80 °C (Figure 1c) [22]. For the photo-polymerization of the photonic gel, 1.25 g of HEMA, 0.075 g of MBAA, 1.25 g of the NIPAAM/ACMO mixture with five different molar ratios of HEMA:MBAA:NIPAAM:ACMO (48:2:50:0, 48:2:35:15, 48:2:25:25, 48:2:15:35, and 48:2:0:50, respectively), and 0.625 g of DI water were premixed. Each mixture was then infiltrated individually into one of the five channels of the DEECA cell, in order to fill the interstice of each opal template without disassembling the cell (Figure 1d). The photo-polymerization was then performed by exposing the cell to a UV light source (SB-100P/F) for 60 min (Figure 1e). After the cell was disassembled, it was soaked in chloroform for 24 h in order to etch away the PS opal templates (Figure 1f). After rinsing in an intermediate solvent of acetonitrile (MeCN) several times, the photonic gels were transferred through a water bath circulator to a custom-made metal dish containing thermostated DI water. The actual temperature of each photonic gel was measured using a temperature probe soaked in DI water. During each measurement, the metal dish was capped with a concave watch glass that touched the water in order to enable a visualization of the thermochromic photonic gel in a wide range of temperatures. The temperature varied from 10 to 80 °C, and an optical measurement was performed after the thermochromic gel had reached a desired temperature (typically after 30 min). At each equilibrium temperature, the optical photograph of a photonic gel was taken by a digital camera (DSLR-A550, Sony, Tokyo, Japan). The reflectance spectrum of the gel was then obtained by using a fiber-optic UV-Vis spectrometer (AvaSpec, Avantes, Apeldoorn, The Netherlands) coupled with a 10× objective lens (N.A. = 0.4) used by a reflected light microscope (L2003A, Bimeince). Each spectrum was noted through the use of a circular aperture (area ~1 mm^2^) with a solid angle of ~10°, so that near-specular reflection could be assumed. A silver mirror (Edmund Optics, Barrington, NJ, USA) was used as a reflectance reference. The structures of the dried photonic gels were imaged before and after template removal by using a scanning electron microscope (SEM) (S-4700, Hitachi, Tokyo, Japan). 

## 3. Results and Discussion

**Figure 2:**
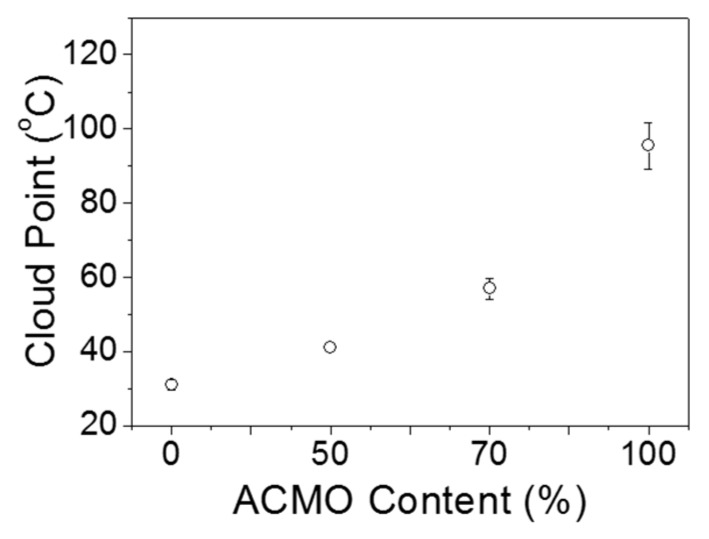
Cloud point measurements of PACMO-PNIPAAM copolymers with four different monomer ratios. The values from multiple measurements were averaged and plotted.

In this study, a variation in the LCST was anticipated due to the copolymerization of ACMO and NIPAAM, which exhibit LCSTs of 88 °C and 33 °C, respectively, in aqueous systems. In order to verify this hypothesis, radical copolymerizations of PACMO-PNIPAAM were undertaken at four different ACMO contents (0, 50, 70, and 100 wt %), with each using azobisacrylonitrile (AIBN) as an initiator. The four copolymers were individually dissolved in water at 10 wt % concentration, and the cloud points were measured (see (Figure S1) for some examples). The average cloud points are plotted in Figure 2, which shows the elevation of the LCST of the copolymer with the increased ACMO contents. 

**Scheme 1:**
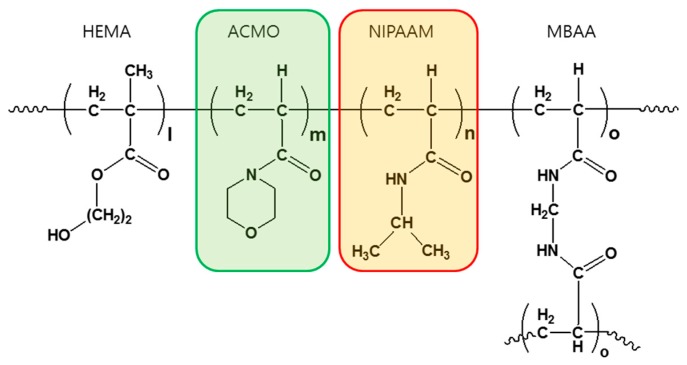
The molecular structure of a thermochromic gel consisting of HEMA as a hydrogel building block, ACMO and NIPAAM as the thermochromic gels, and NBAA as a crosslinker, which are random-copolymers with molar ratios of l:m:n:o.

When it came to fabricating the thermochromic photonic gels, the five monomer mixtures containing different ratios of HEMA, ACMO, NIPAAM, and MBAA were infiltrated into five channels of the DEECA, all containing the same opal template, which is shown in Figure 1d. A UV photo-polymerization formed the crosslinked copolymeric hydrogels—their molecular structures can be seen in Scheme 1. 

**Figure 3:**
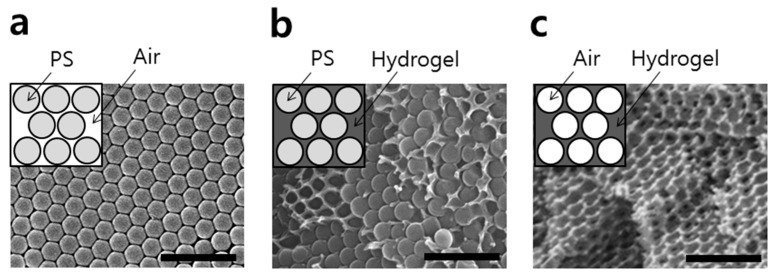
SEM images of a (**a**) PS opal template, (**b**) opal template with a cross-linked thermochromic gel, and (**c**) a thermochromic inverse opal gel after template removal. Scale bars in the images represent 1 µm.

In order to obtain different temperature-driven swelling responses, the molar ratios of ACMO and NIPAAM in a polymerization mixture (m:n) were controlled to be 50:0, 35:15, 25:25, 15:35, and 0:50, respectively. In this study, we fabricated the periodic structure via the opal-templated photopolymerization of the hydrogel. An opal-templated photonic gel exhibits a periodic modulation of the refractive indices within a gel, and an appropriate periodic distance allows it to exhibit a specific color due to the Bragg diffraction from the periodic inverse opal (IO) structure. Figure 3a shows a PS opal template fabricated by the flow-cell method [23]. 

Figure 3b shows a thermochromic gel that is polymerized within the interstitial spaces of an opal template. As shown in Figure 3c, upon removal of the PS template the porous IO structure of the thermochromic photonic gel was obtained. Self-assembled opal films usually expose the (111) planes of a face-centered cubic (FCC) parallel to the surface of a substrate, from which the Bragg diffraction of incident light takes place. The peak wavelength (λ_peak_) of diffracted light from an opal or an IO film is approximately related to the diameter of the templating particle (*d*) by the modified Bragg formula that is shown in Equation (1) [23].

(1) $$\begin{array}{cc} \lambda_{\mathrm{peak}} & =\;{\left(8/3\right)}^{1/2}\cdot d\cdot {\left(f_{pore}\cdot n_{pore}{}^{2}+f_{gel}\cdot n_{gel}{}^{2}-{\;\sin}^{2}\theta \right)}^{1/2}\\ & =\;1.633\cdot d\cdot {\left(f_{pore}\cdot n_{pore}{}^{2}+f_{gel}\cdot n_{gel}{}^{2}\right)}^{1/2};\;\mathrm{at}\;\theta =\;0\\ & =\;1.633\cdot d\cdot n_{eff} \end{array}$$

In this formula: *d* is the pore size of the IO structure; *n_pore_*, *n_gel_*, *f_pore_*, and *f_gel_* are the refractive indices and filling factors of the pore and the gel, respectively; and *θ* is the angle measured from the normal to the opal film surface, which is approximately 0°. One can estimate that the *n_eff_* of a HEMA-based hydrogel in water is ~1.38 [17].

**Figure 4:**
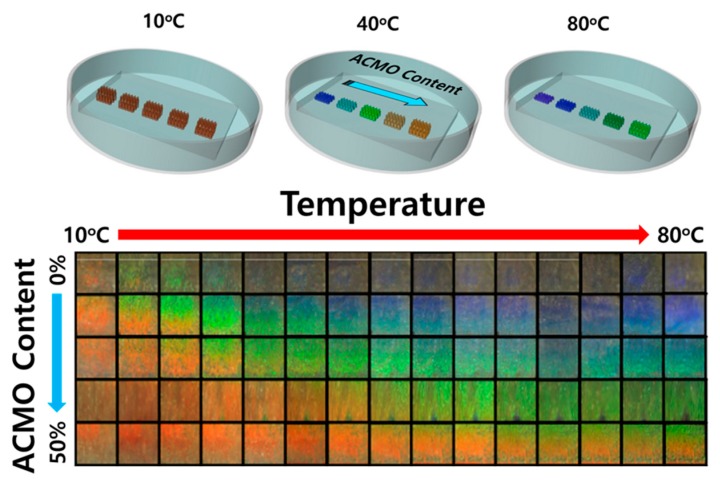
The temperature-driven color changes of five thermochromic photonic gels whose ACMO contents were 0, 15, 25, 35, and 50 mol %, respectively. Five photonic gels were templated by the same opal arrays on a single substrate. The time interval for each temperature was 30 min. As the ACMO content increased in the gel, the reflective colors tended to red-shift at a given temperature due to the more swollen state of the gel.

**Figure 5:**
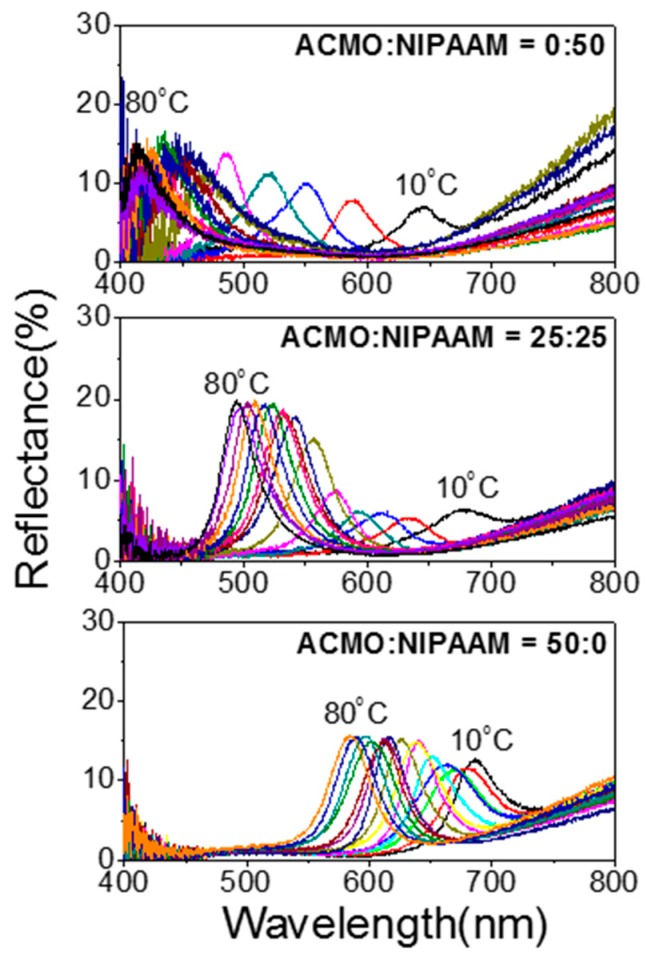
Temperature-dependent changes of the reflectance spectra of three IO photonic gels containing different molar ratios of NIPAAM and ACMO.

After the optimized procedure, the thermochromic gel templated by PS-230 exhibited a red reflective color in a water bath temperature-stabilized at 10 °C; this occurred as the temperature of the bath was far below the LCST of both polymers. Therefore, the gel was in a fully swollen state regardless of the NIPAAM and ACMO contents. The molar ratio of HEMA, the temperature-responsive monomers, and the crosslinker was maintained at 48:50:2, and only the ratios between NIPAAM and ACMO were varied. The thermochromic responses of the five photonic gels were obtained by photographic measurements of their color changes (Figure 4) and also by spectroscopic reflectance measurements (Figure 5). 

In Figure 4, the temperature-dependent color changes of the five photonic gels templated by PS-230 were measured using a digital camera within the temperature ranges of 10–80 °C at every 5 °C interval. In the first row of Figure 4 are the temperature-dependent color changes of the NIPAAM-only gel (NIPAAM:ACMO = 50:0), in which the temperature variation gave rise to the color changes in the photonic gel sensor for the entire visible light spectra. Changes in the reflective colors above 30 °C are not, however, readily distinguishable due to the negligible color changes that occur for temperatures above the LCST of NIPAAM. However, incorporation of ACMO enabled clearly visible color changes of the thermochromic gel to occur above 50 °C, as shown in Figure 4. The photonic gel with a molar ratio of NIPAAM:ACMO = 25:25 brought about more distinguishable color changes from red to blue as a thermochromic gel at the given temperature ranges. The photonic gel of NIPAAM:ACMO = 0:50 exhibited only red colors at low temperature ranges, owing to the highly swollen states of the gels, but the color changed from red to green with temperature elevation. Compared to a visualization of color change with temperature alteration, a reflectance measurement shows the variation of photonic stopband positions (λ_peak_) at each temperature in a more precise and quantitative way. Figure 5 shows the changes of the temperature-dependent reflectance spectra of the thermochromic gels with varying ACMO content. A quantitative measurements of λ_peak_ enable the estimation of varying pore size of the inverse opal hydrogel. According to Equation (1), a longitudinal distance between pores (*d*) can be approximated as λ_peak_/(1.633*n_eff_*) assuming *n_eff_* ~1.38 for swollen hydrogel. For a photonic gel sensor with NIPAAM:ACMO = 25:25, since λ_peak_ values at 80 °C and 10 °C are 490 nm and 675 nm, respectively, then *d* can be estimated respectively to be 217 nn and 299 nm.

The color changes and the spectral changes can also be tuned by using different template particle sizes. In Figure S2, those data obtained from the photonic gels are templated by PS-235, which has a larger particle diameter.

**Figure 6:**
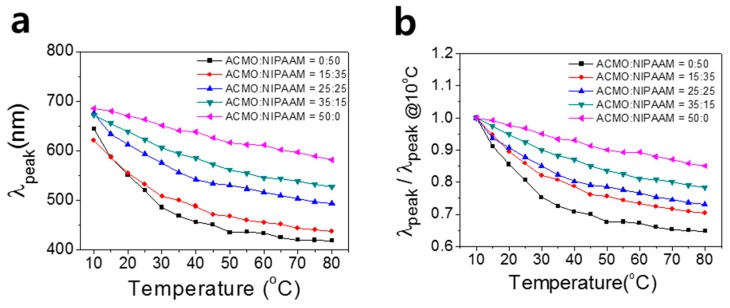
(**a**) Plot of the stopband positions (λ_peak_) of five different IO photonic gels vs. temperature; (**b**) Plot of λ_peak_ normalized to that of 10 °C with temperature ranges of 10–80 °C.

During temperature rise, λ_peak_ for each sensor was obtained from each spectrum and plotted as a function of temperature, as shown in Figure 6a. Furthermore, λ_peak_ was normalized to the value of the lowest temperature for each sensor and plotted, as shown in Figure 6b. In Figure 6a, temperature variations from 10 to 80 °C for a thermochromic gel with 50% NIPAAM (0% ACMO) brought about a change in λ_peak_ from 645 nm to 415 nm, with a steeper slope (dλ_peak_/dT) for the low temperature regime. A photonic gel with 100% ACMO, however, exhibited a less steep slope (dλ_peak_/dT) but had linear temperature-driven spectral changes at the temperature ranges of 10–80 °C. The distinct slopes in the T-dependent λ_peak_ positions from the five thermochromic gels originate from the different LCSTs of NIPAAM and ACMO. Since the LCST of ACMO is higher than that of NIPAAM, the shrinkage of the ACMO hydrogel with temperature rise occurs at a higher temperature than that of the NIPAAM gel. For this reason, higher ACMO content in the photonic gel exhibited steeper at high temperature region. Particularly at 70–80 °C temperature ranges in Figure 6, the dλ_peak_/dT values of photonic gels with three different ACMO content (0%, 25%, 50%) were calculated to be 0.8 nm/°C, 1.8 nm/°C, and 2.5 nm/°C respectively.

The results shown in Figure 6 imply that the color tunability of the thermochromic sensor can be easily controlled by changing the mixing ratio of ACMO and NIPAAM, and the temperature sensing range can be higher than 50 °C. There have been reports of thermochromic photonic sensors using NIPAAM, but the detection of temperature change was limited to 40 °C due to its low LCST (~33 °C). The thermochromism of the photonic gel sensors was investigated up to 80 °C, above which it both took a long time for the temperature to reach an equilibrium, and a bubble started to form inside the photonic gel. 

**Figure 7:**
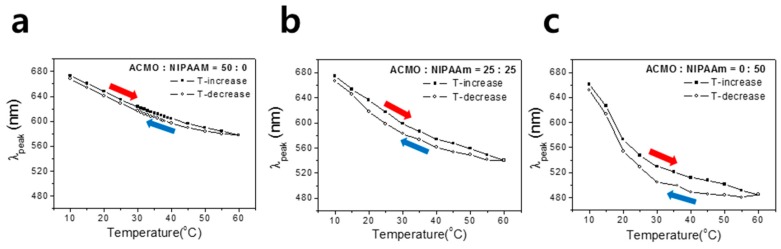
Hysteresis plots of the temperature-dependent stopband positions (λ_peak_) of three different IO photonic gels with relative ACMO contents of (**a**) 100%, (**b**) 50%, and (**c**) 0%. Heating and cooling rate was set as 5 °C/min, and equilibrated for 30 min at each designated temperature before optical measurements.

The reproducibility of the thermochromic gel was examined by monitoring λ_peak_ changes during the heating and cooling cycles. The temperature of the bath containing a thermochromic gel was increased from 10 °C to 60 °C and equilibrated for 30 min at each designated temperature for the λ_peak_ measurement, for which closed square plots were made. When the temperature reached 60 °C, the cell was cooled down and measurements were taken at each temperature point for λ_peak_ (shown as open circle). As shown in Figure 7a, the thermochromic gel with NIPAAM:ACMO = 0:50 showed fairly well-matched photonic stopbands for heating and cooling, while the gel with the increased NIPAAM content brought about significant hysteresis; this was presumably due to a delayed restoration of the shrunken IO gel to the swollen state in spite of the previously-reported fast kinetics of an IO thermochromic photonic gel on temperature change [14]. In a shrunken IO structure, the influx of water is often delayed due to the narrowed pathways connecting the pores [23]. As a comparison, Δλ_peak_ (λ_peak,cooling_ − λ_peak,heating_) for the three thermochromic gels at 30 °C were 1%, 4%, and 6%, as shown in Figure 7c.

## 4. Conclusions

Thermochromic photonic gels containing NIPAAM and ACMO were investigated, which respectively have LCSTs of 33 °C and ~95 °C in water. By varying the temperature from 10 °C to 80 °C, changes in the λ_peak_ of five thermochromic gels with different ACMO contents were noted, and it was found that the ACMO-containing thermochromic gels exhibit more uniform dλ_peak_/dT in the given temperature range as well as a smaller hysteresis of λ_peak_ on the heating and cooling cycle in comparison to a NIPAAM-only gel. 

## Supplementary Materials

The following are available online at http://www.mdpi.com/1424-8220/17/6/1398/s1, Figure S1: Examples of photographs showing the cloud point measurements, Figure S2: Color changes and selected spectral changes of thermochromic photonic gels with different ACMO contents templated by PS-235.
