# Supplementary figures and images for: Tunable Temperature Response of a Thermochromic Photonic Gel Sensor Containing N-Isopropylacrylamide and 4-Acryloyilmorpholine
Source: Sensors (Basel). 2017 Jun 15;17(6):1398. doi: 10.3390/s17061398 (PMC5492457; doi:10.3390/s17061398)

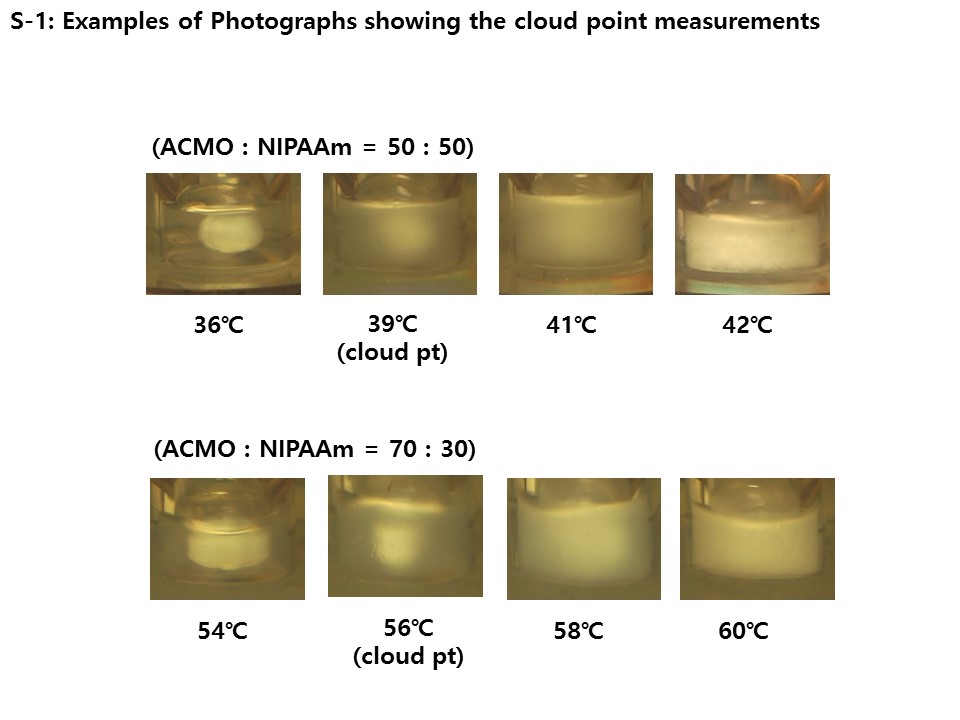

Supplement: Supplementary file 1 [file sensors-17-01398-s001.zip › Figures Nipam-ACMO sensor-Suppl/S-1.JPG]

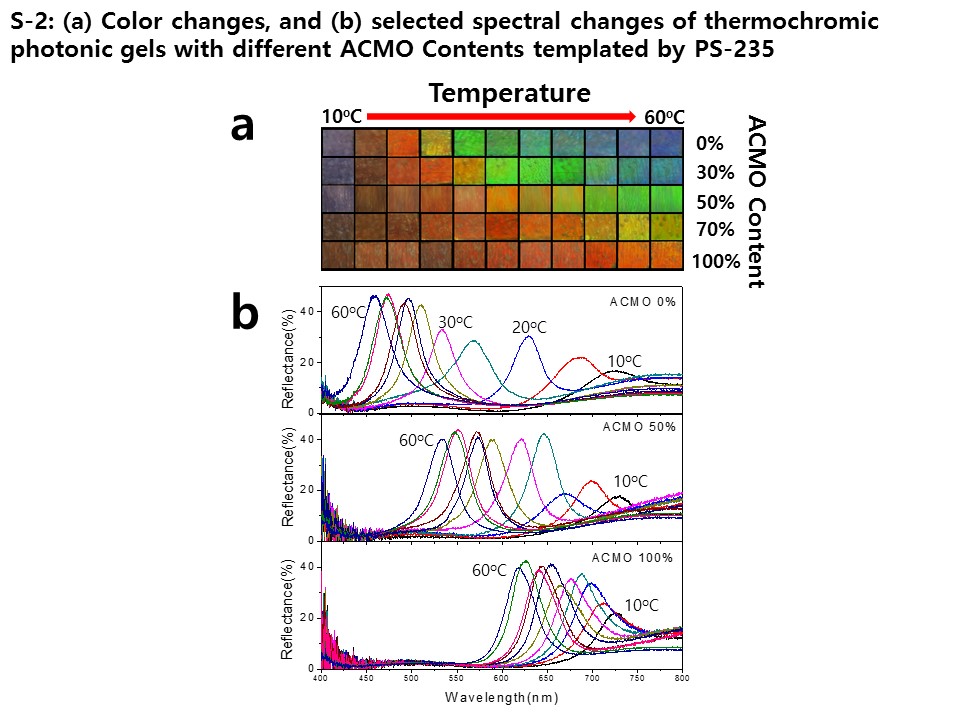

Supplement: Supplementary file 1 [file sensors-17-01398-s001.zip › Figures Nipam-ACMO sensor-Suppl/S-2.JPG]
